# Genome-Wide Identification of WRKY Gene Family in Artemisia and Its Expression Analysis of Aphid Resistance

**DOI:** 10.3390/ijms27072981

**Published:** 2026-03-25

**Authors:** Lanjie Xu, Sufang An, Qing Yang, Xiaohui Wu, Hongqi Yang, Junping Feng, Yazhou Liu, Zhansheng Nie, Yongliang Yu, Huizhen Liang

**Affiliations:** 1 Institute of Chinese Herbal Medicines, Henan Academy of Agricultural Sciences, Zhengzhou 450002, China; xulanjie18@126.com (L.X.); ansufang888@163.com (S.A.); qingyang316@126.com (Q.Y.); wuxiaohui8688@163.com (X.W.); yang13303833929@163.com (H.Y.); junpingfeng123@163.com (J.F.); yzhoul@126.com (Y.L.); nzs3911@126.com (Z.N.); 2 Provincial Key Laboratory of Conservation and Utilization of Traditional Chinese Medicine Resources in Henan, Zhengzhou 450002, China

**Keywords:** WRKY, expression analysis, isoflavonoid biosynthetic, biotic stress

## Abstract

WRKY is a crucial transcription factor involved in plant growth, development, and responses to abiotic stress. In the present study, a total of 182 *AaWRKY* transcription factor members were identified across the *Artemisia argyi* genome and found to be distributed across 17 chromosomes. Evolutionary analysis revealed that segmental duplication served as the primary driver for family expansion, with the evolutionary trajectory shaped by strong purifying selection (Ka/Ks < 1.0). Phylogenetic classification categorized these members into seven highly conserved subgroups, while physicochemical analysis indicated that most AaWRKYs are unstable, hydrophilic proteins, consistent with the rapid turnover required for transcriptional switches. Transcriptomic profiling unveiled significant tissue-specific expression patterns, with over 50% of the members predominantly enriched in roots and specific genes, such as *AaWRKY11*, implicated in the regulation of leaf senescence. Protein–protein interaction (PPI) network analysis identified AaWRKY110 as a central regulatory hub linking the MAPK signaling pathway with the isoflavonoid biosynthetic machinery. Furthermore, comparative transcriptomic analysis between aphid-resistant (Ai20K) and susceptible (Ai72G) cultivars demonstrated that resistance is conferred by a priming mechanism involving high basal expression of key candidates, including *AaWRKY82*, *108*, *128*, and *71*. In contrast, the susceptible genotype exhibited a delayed and ineffective hypersensitive-like response. Collectively, these findings elucidate the evolutionary dynamics of the *AaWRKY* family and provide critical genetic targets for the concurrent improvement of medicinal metabolite accumulation and biotic stress resilience in *Artemisia argyi* via molecular breeding.

## 1. Introduction

Plant transcription factors are a group of proteins that possess DNA-binding domains and play a role in regulating gene expression. By binding to specific DNA sequences, they can influence the binding of RNA polymerase and either activate or inhibit gene transcription [1]. Common transcription factors found in plants include bHLH, bZIP, NAC, WRKY, MYB, and GATA. These factors are involved in regulating various biological processes in plants, such as growth and development, the production of secondary metabolites, disease resistance, and responses to environmental stress [2,3,4,5,6,7].

The WRKY transcription factor family is one of the largest regulatory factor families in the genomes of higher plants [8]. Its name is derived from the highly conserved WRKY domain in its protein sequence. This domain typically contains around 60 amino acid residues, with the core feature being a WRKYGQK motif composed of seven amino acids at the N-terminus, followed by a zinc finger structure (Zinc-finger motif) formed by cysteine and histidine at the C-terminus [9]. WRKY transcription factors primarily regulate downstream gene transcription levels by embedding their highly specialized domain, resembling a “β-wedge” shape, into the major groove of the target gene promoter’s W-box DNA [10]. The members of this family are rigorously categorized into three groups: Group 1, Group 2, and Group 3, based on the number of WRKY domains and the specific type of zinc finger motifs. Although the WRKYGQK sequence is considered the “signature” of this family, it has undergone diverse natural mutations over long-term evolution to adapt to changing environments [11]. These variations include WRKYGKK, WKKYGQK, WRKYGEK, WRKYGRK, and even incomplete deletions. Studies have shown that substitutions of key residues (such as mutations at positions 5 or 6) often result in a reduced binding affinity between the transcription factor and the W-box, or enable the factor to bind to other cis-regulatory elements, thus expanding the regulatory landscape of the WRKY family [12]. Such variations are especially common in Asteraceae plants (sunflower and Artemisia species), suggesting that these plants optimize their defense responses by modulating transcription factor affinity when adapting to specific biotic and abiotic stresses. With the release of more information on plant genomes, WRKY family genes have been successfully identified in many species, such as *Vaccinium bracteatum* [13], *Lycium barbarum* [14], *Amaranthus hypochondriacus* [15], *Leonurus japonicus* [16], *Miscanthus sinensis* [17], *Cucurbita pepo* [18], *Rosa chinensis* [19], *Glycyrrhiza uralensis* [20], *Carthamus tinctorius* [21] and so on.

WRKY transcription factors are reported to be extensively involved in plant growth and development. They serve as important regulators of multiple biological processes, including nitrogen metabolism, stress response, flowering, and hormone signaling. Emerging evidence indicates that WRKY transcription factors (TFs) serve as multifaceted regulators of plant physiological processes [8]. Numerous WRKY members bolster plant resilience against drought, salinity, extreme temperatures, and heavy metal toxicity by modulating osmotic homeostasis or fortifying the reactive oxygen species (ROS) scavenging system. Specifically, *CsWRKY7* in Camellia sinensis improves cold tolerance by tuning abscisic acid (ABA) sensitivity [22]. In Arabidopsis thaliana, *AtWRKY33* functions as a central hub for thermotolerance by orchestrating the expression of heat shock proteins (HSPs) [23], whereas *AtWRKY13* is critical for mitigating cadmium (Cd) toxicity. Beyond stress responses [24], WRKY TFs are integral to nutrient acquisition and developmental programming. *AtWRKY75* was the inaugural member identified to mediate the phosphate (Pi) starvation response, directly influencing nutrient uptake via the transcriptional regulation of phosphate transporters [25]. In developmental contexts, *MdWRKY71* in *Malus domestica* accelerates the floral transition by activating floral meristem-specific genes [26]. In Arabidopsis, *AtWRKY53* acts as a hallmark regulator of leaf senescence, precisely modulating programmed cell death during nutrient remobilization through complex signaling crosstalk [27]. Notably, in *Artemisia* species, WRKY TFs are indispensable for the initiation of glandular secretory trichomes (GSTs). *AaGSW1*, for instance, integrates light and phytohormone signaling to drive the formation of these specialized “bio-factories” where artemisinin and volatile oils are synthesized [28]. Furthermore, WRKY TFs play a decisive role in the metabolic engineering of medicinal plants. *SmWRKY1* significantly enhances tanshinone accumulation in *Salvia miltiorrhiza* by upregulating multiple key biosynthetic enzymes [29]. Meanwhile, *CrWRKY1*’s involvement in terpenoid indole alkaloid (TIA) biosynthesis in *Catharanthus roseus* underscores the therapeutic potential of utilizing WRKY TFs to optimize the production of potent anticancer metabolites [30].

*Artemisia argyi*, a prominent perennial herb, holds profound medicinal and cultural significance in East Asia [31]. The plant’s pharmacological efficacy—comprising anti-inflammatory, analgesic, and antimicrobial activities—is attributed to an abundance of secondary metabolites such as volatile oils, flavonoids, and terpenoids, making it an indispensable resource for both traditional moxibustion and modern phytochemical industries. The recent assembly of the *Artemisia argyi* genome has shifted research focus toward deciphering the complex regulatory circuits that dictate metabolite accumulation and stress resistance. While the WRKY transcription factor (TF) family is well-documented as a master regulator in other species, there is a notable scarcity of systematic research addressing the WRKY family at the whole-genome level in *Artemisia argyi*, especially concerning its involvement in isoflavone biosynthesis and defense against insect pests. This study leverages genomic data to systematically characterize the *Artemisia argyi* WRKY family, integrating phylogenetic analysis, structural characterization, and cis-element prediction with spatio-temporal expression profiling. Our results not only provide a framework for understanding the transcriptional control of secondary metabolism but also pave the way for the molecular improvement of *Artemisia argyi* varieties with enhanced phytochemical profiles and robust herbivory resistance.

## 2. Results

### 2.1. Identification and Physicochemical Properties of the WRKY Gene Family in Artemisia argyi

**Figure 1:**
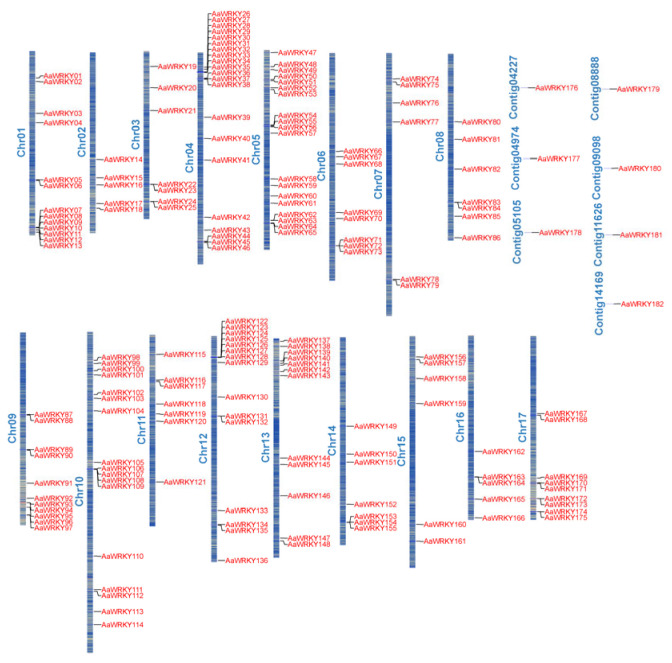
Genomic localization and distribution patterns of the *AaWRKY* family. The ideogram illustrates the physical mapping of 182 *AaWRKY* genes. Red labels denote the specific loci of WRKY members, while blue labels identify the 17 chromosomes and 7 unanchored contigs. The gradient/shading within the chromosomal bars represents the overall gene density across the *Artemisia argyi* genome. Genes located on the 7 unplaced scaffolds (contigs) are shown separately to indicate their non-anchored status in the current genome assembly. In the figure, the blue color on the chromosomes represents high gene density, while the yellow color represents low gene density.

A genome-wide localization analysis revealed that the 182 identified *AaWRKY* members are distributed across 17 chromosomes and 7 unplaced contigs (Figure 1, Table S1). The distribution was non-random; Chromosome 4 exhibited the greatest abundance of *AaWRKY* genes (21 members), while Chromosomes 2 and 16 showed the minimum coverage, with 5 members each. Furthermore, the remaining 7 *AaWRKY* genes were individually anchored to separate contigs. This heterogeneous distribution suggests potential gene expansion or loss events during the evolution of the *Artemisia argyi* genome. Systematic analysis of the physicochemical properties of the 182 identified *Artemisia argyi* WRKY (AaWRKY) proteins revealed substantial molecular diversity (Figure S1). The protein sequences ranged from 124 (AaWRKY38) to 894 amino acids (AaWRKY104) in length, with an average of 344.72 aa, and corresponding molecular weights spanning from 14.24 to 101.55 kDa (average 38.53 kDa). The theoretical isoelectric points (*pI*) varied widely from 4.78 to 10.11, consisting of 105 acidic (*pI* < 7.0) and 77 basic (*pI* > 7.0) members, suggesting functional adaptability across diverse cellular pH environments. Stability analysis showed that the instability index ranged from 30.22 to 76.16, with a mean of 50.12; specifically, 92.9% of the members (169 proteins) were predicted to be unstable, while only 7.1% (13 proteins) were classified as stable, highlighting the rapid turnover nature typical of transcription factors in response to environmental stimuli. Furthermore, the grand average of hydropathicity (GRAVY) values for all AaWRKY proteins were negative, ranging from −1.187 to −0.413 (average −0.814), indicating that all members are hydrophilic in nature, which is consistent with their biological role in the aqueous environment of the nucleus. The aliphatic index ranged from 40.68 to 85.45, with an average value of 60.16.

### 2.2. Phylogenetic Analysis and Classification of the AaWRKY Family

**Figure 2:**
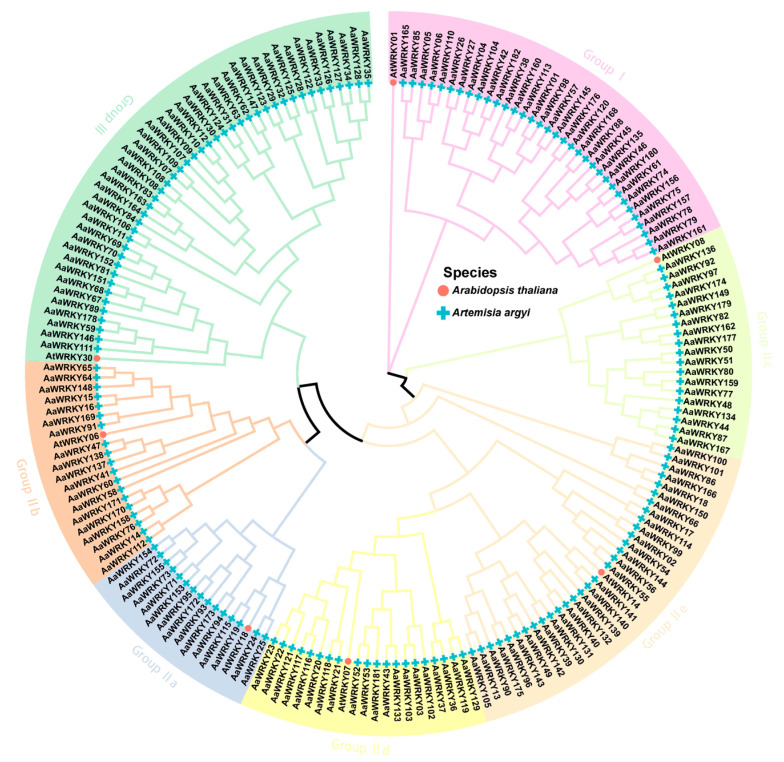
Phylogenetic analysis and classification of WRKY proteins from *Artemisia argyi* and *Arabidopsis thaliana*. The circular phylogenetic tree was constructed based on the full-length protein sequences of 182 AaWRKYs and representative AtWRKYs using the Maximum Likelihood (ML) method. The WRKY members are categorized into three primary groups (Group I, II, and III) and five Group II subgroups (IIa, IIb, IIc, IId, and IIe), which are distinguished by different background colors. Red circles and blue plus signs represent *Arabidopsis thaliana* and *Artemisia argyi* WRKY members, respectively. The branch lengths represent evolutionary distances.

The 182 WRKY genes in mugwort were systematically categorized by constructing a phylogenetic tree, incorporating seven well-characterized members of *Arabidopsis thaliana*: *AtWRKY01*, *AtWRKY06*, *AtWRKY08*, *AtWRKY14*, *AtWRKY07*, *AtWRKY18*, and *AtWRKY30*, in conjunction with the WRKY genes from mugwort (Figure 2). The resulting phylogenetic tree, depicted in Figure 2, distinctly classifies the WRKY genes in mugwort into seven discernible groups: Group I, Group IIa, Group IIb, Group IIc, Group IId, Group IIe, and Group III. Group I encompasses 34 genes, Group IIa comprises 15 genes, and Group IIb consists of 19 genes. Furthermore, Group IIc and Group IId each contain 19 and 20 genes, respectively, while Group IIe encompasses 31 genes, and Group III comprises 44 genes. The classification of the WRKY gene in mugwort mirrors that observed in the model organism Arabidopsis thaliana, suggesting that the WRKY gene is conserved across various plant species.

### 2.3. Syntenic Characterization and Evolutionary Drivers of the AaWRKY Family

**Figure 3:**
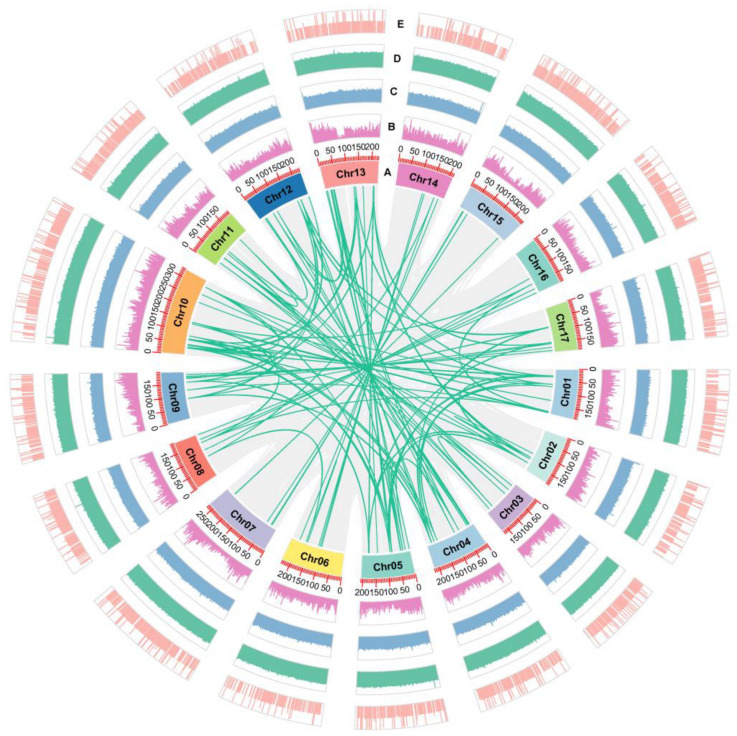
Genomic Distribution and Evolutionary Expansion of the *AaWRKY* Family. The Circos plot illustrates the genomic landscape and duplication patterns of the WRKY family. The various tracks represent the following features: (A) The 17 chromosomes of the *Artemisia argyi* genome, with scales indicated in megabases (Mb); (B) Global gene density along each chromosome; (C) N-ratio distribution; (D) GC-ratio distribution; (E) GC-skew profile. The green lines in the center of the plot represent the syntenic *AaWRKY* gene pairs, highlighting the segmental duplication events within the genome.

To elucidate the evolutionary trajectory and expansion patterns of the *AaWRKY* family, we conducted a comprehensive collinearity analysis across the *Artemisia argyi* subgenomes. Our findings revealed 139 syntenic gene pairs, predominantly arising from segmental duplication events (Figure 3, Table S2). The prevalence of segmental over tandem duplications indicates that large-scale genomic rearrangements and whole-genome duplication (WGD) events were the fundamental forces driving the numerical expansion of WRKY members. To further assess the nature of the selection pressure acting on these duplicated pairs, we calculated the nonsynonymous to synonymous substitution ratios (Ka/Ks). Most *AaWRKY* gene pairs exhibited Ka/Ks ratios of less than 1.0 (Figure S2), suggesting that this family has undergone strong purifying selection to maintain functional stability throughout its evolutionary history.

### 2.4. The Collinearity Analysis of WRKY Family Genes Between Artemisia argyi and Other Plants

**Figure 4:**
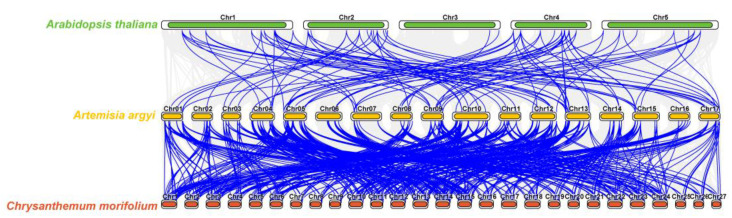
Syntenic analysis of WRKY genes between *Artemisia argyi* and two representative species. The gray lines in the background represent the collinear blocks between the genomes of *Artemisia argyi* and other species (*Arabidopsis thaliana* and *Chrysanthemum morifolium*). The blue lines highlight the syntenic (orthologous) WRKY gene pairs. The species names and their corresponding chromosomes are labeled on the left and above each genomic track, respectively. The top, middle, and bottom tracks represent the chromosomes of *A. thaliana*, *Artemisia argyi*, and *C. morifolium*, respectively.

To elucidate the genomic conservation and evolutionary trajectory of the WRKY transcription factor family, we constructed comparative syntenic maps between *Artemisia argyi* and two representative genomes: the model dicot *Arabidopsis thaliana* and the fellow Asteraceae member *Chrysanthemum* (Figure 4). The analysis revealed extensive collinearity, identifying 106 and 629 orthologous WRKY gene pairs between *Artemisia argyi* and *A. thaliana*, and *Artemisia argyi* and *Chrysanthemum*, respectively. Notably, *Artemisia argyi* exhibited a significantly higher degree of syntenic conservation with *Chrysanthemum* than with *A. thaliana*. This enhanced collinearity reflects the close evolutionary proximity of species within the Asteraceae family and suggests that numerous WRKY members have been retained through purifying selection since their last common ancestor. Furthermore, the identification of these orthologous pairs facilitates the functional annotation of *AaWRKY* genes, particularly those involved in secondary metabolism and stress responses that are conserved within the Asteraceae lineage.

### 2.5. Protein–Protein Interaction (PPI) Network Analysis of AaWRKYs

**Figure 5:**
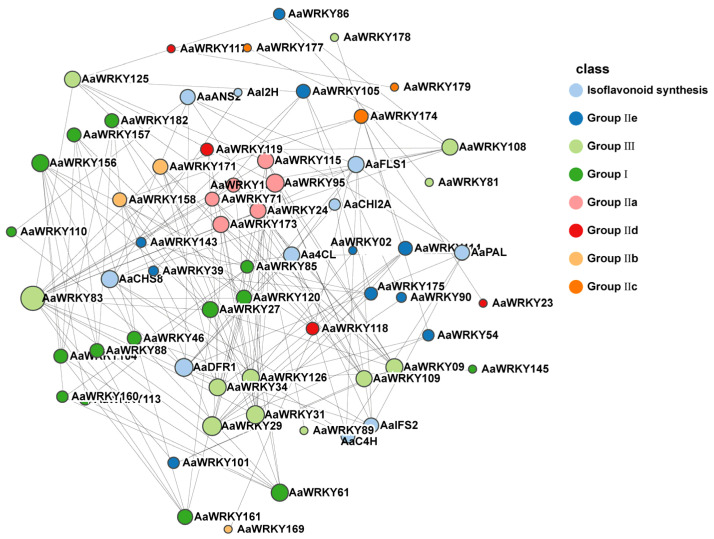
Predicted protein–protein interaction (PPI) network of AaWRKYs and isoflavonoid biosynthetic enzymes. This interactome illustrates the potential regulatory relationships between AaWRKY proteins and key enzymes in the isoflavonoid biosynthetic pathway within *Artemisia argyi*. The network was constructed using the STRING database based on genomic context, co-expression data, and functional associations. Different colors represent different groups of WRKY proteins.

To predict the potential functions of AaWRKY proteins, a protein–protein interaction (PPI) network was constructed using the STRING database. Among the 182 identified members, 54 AaWRKYs were found to be involved in the interaction network, representing all seven phylogenetic subgroups (Figure 5). Notably, the network also incorporated 10 key enzymes associated with isoflavone biosynthesis, including Aa4CL, AaANS2, AaC4H, AaCHI2A, AaCHS8, AaDFR1, AaFLS1, AaIFS2, AaPAL, and AaI2H. Within this interactome, *AaWRKY110* emerged as a central hub, exhibiting extensive interactions with the majority of the isoflavone biosynthetic proteins. These findings suggest that AaWRKY proteins may play a regulatory role in the isoflavone biosynthetic pathway, which is likely linked to plant growth, development, and responses to biotic stress in *Artemisia argyi*.

### 2.6. Tissue-Specific and Age-Dependent Expression Profiles of AaWRKY Genes

**Figure 6:**
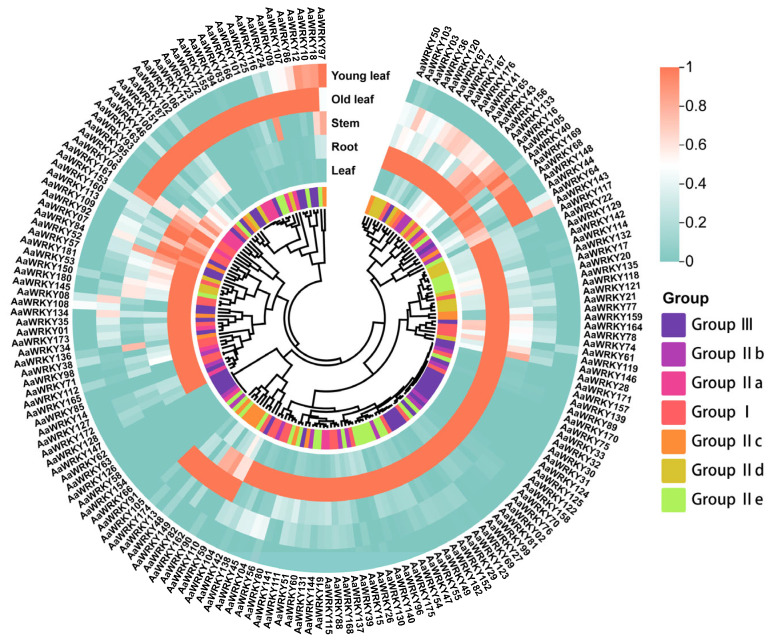
Expression profiles of *AaWRKY* genes across different tissues of *Artemisia argyi*. The central dendrogram represents the clustering of genes based on the similarity of their expression profiles across the sampled materials. Each radial line represents an individual *AaWRKY* gene, while the concentric rings correspond to the different tissue types. The color gradient reflects Z-score normalized expression values, ranging from blue (low expression, 0) to red (high expression, 1.0). The map highlights distinct groups of genes with tissue-specific accumulation. For instance, certain clusters show preferential expression in the roots or leaves, suggesting functional specialization of these transcription factors during the growth and development of mugwort.

The expression analysis of 178 *AaWRKY* genes across five distinct tissues. root, stem, leaf, old leaf, and young leaf unveiled a highly heterogeneous and tissue-biased regulatory landscape. Our results demonstrate that the root is the primary site of *AaWRKY* activity, with 95 members (approximately 52.5% of the family) exhibiting peak expression levels in this organ (Figure 6). This root-dominant cluster primarily includes members from Group IIb (*AaWRKY15*, *AaWRKY47*, and *AaWRKY170*) and specific members from Group III (*AaWRKY70* and *AaWRKY69*), suggesting their pivotal roles in root development, nutrient acquisition, and the perception of soil-borne environmental cues. In the aerial parts, 38 and 34 *AaWRKY* genes showed preferential expression in the Leaf and Old leaf, respectively, while only one gene reached its maximum expression in the young leaf. Notably, several Group III members (*AaWRKY35* and *AaWRKY128*) and Group I members (*AaWRKY01* and *AaWRKY38*) were highly enriched in mature leaves, potentially orchestrating the biosynthesis of specialized metabolites such as terpenoids and flavonoids. The distinct expression shift observed between “Leaf” (mature) and “Old leaf” (senescing), exemplified by genes like *AaWRKY11*, indicates that specific WRKY subfamilies have undergone functional specialization to regulate leaf senescence and nutrient recycling. Conversely, the minimal expression of *AaWRKYs* in the stem and young leaf suggests a more specialized or stress-inducible requirement in these tissues. Combined with the evolutionary evidence of strong purifying selection (Ka/Ks < 1), this broad yet tissue-specific expression distribution underlines the functional diversification of the *AaWRKY* family, enabling *Artemisia argyi* to precisely coordinate its growth, development, and chemical defense across different organs and developmental stages.

### 2.7. Differential Expression Analysis of AaWRKY Genes Across Different Materials

**Figure 7:**
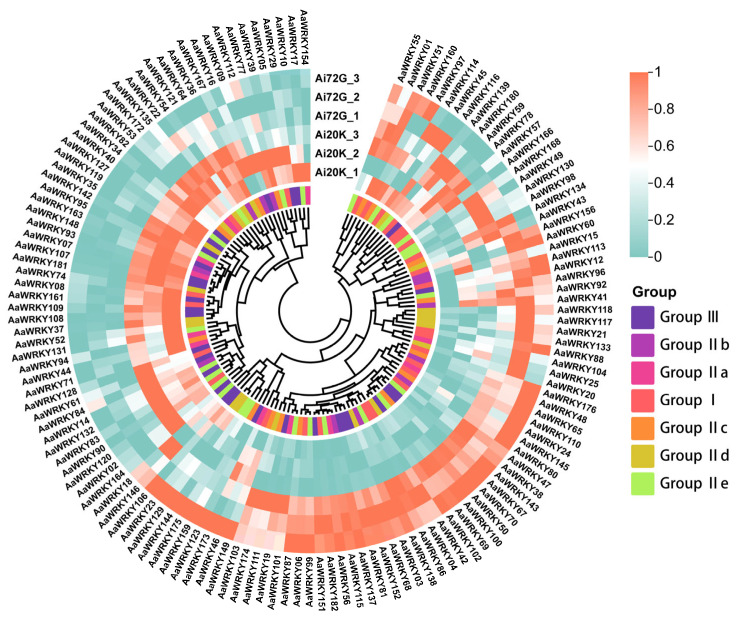
Differential expression analysis of AaWRKY genes between two experimental groups. The columns represent six samples, consisting of three biological replicates for each group: Ai20K and Ai72G. Both genes (rows) and samples (columns) were subjected to hierarchical clustering based on expression similarity, revealing distinct clusters of co-expressed *AaWRKY* genes. The expression levels are represented as standardized Z-scores, with the color gradient ranging from red (1.0), indicating low expression, to blue (0), indicating high expression. A significant number of *AaWRKY* genes exhibit contrasting expression patterns between the two groups. For instance, a large cluster of genes shows high expression (blue) in the Ai20K group but is suppressed in the Ai72G group, while another cluster displays the opposite trend, suggesting their specific regulatory roles in response to the different conditions or during the developmental stages of *Artemisia argyi*.

To investigate the function of the WRKY gene in mugwort, this study utilized two materials: Ai20K, which exhibits resistance to aphids, and Ai72G, which does not. The results of the transcriptional analysis are presented in Figure S3. Principal component analysis (PCA) demonstrates a clear separation between the two materials, suggesting inherent differences between them. Correlation analysis reveals that the correlation coefficient for samples of the same material is 1, while the coefficients for the other samples exceed 0.8, indicating variability among the mugwort samples. Differential gene analysis identified 6392 down-regulated genes and 6583 up-regulated genes. From these differentially expressed genes, we selected the WRKY gene for further analysis. Expression profiling of 139 *AaWRKY* genes in the aphid-resistant cultivar (Ai20K) compared to the susceptible cultivar (Ai72G) underscored the critical roles of this gene family in mediating biotic stress responses in mugwort (Figure 7). Our findings revealed that the majority of Subgroup IIa and III members exhibited significantly higher basal expression levels in the resistant cultivar Ai20K, indicating their potential function as positive regulators of aphid resistance. Notably, the pronounced expression bias of key candidate genes—including *AaWRKY82* (Group IIc), *AaWRKY108* (Group III), *AaWRKY128* (Group III), and *AaWRKY71* (Group IIa) in Ai20K suggests that these genes may enhance plant defense by activating downstream metabolic regulatory pathways, likely those involved in the biosynthesis of repellent or toxic secondary metabolites. In contrast, the burst of expression in Group I (represented by *AaWRKY110*) and specific Group III members (*AaWRKY151* and *AaWRKY152*) in Ai72G may represent a robust but ineffective hypersensitive response to aphid infestation in susceptible genotypes. Furthermore, the preferential expression of *AaWRKY142* (Group IIe) in Ai20K points to its critical role as a junction in biotic stress signaling, potentially maintaining resistance through precise hormonal crosstalk. Collectively, the functional specialization of *AaWRKY* subfamilies in aphid defense provides a theoretical foundation for enhancing stress resilience and metabolic traits in *Artemisia argyi* through molecular breeding and genetic engineering.

### 2.8. GO and KEGG Enrichment Analysis of AaWRKY Genes

**Figure 8:**
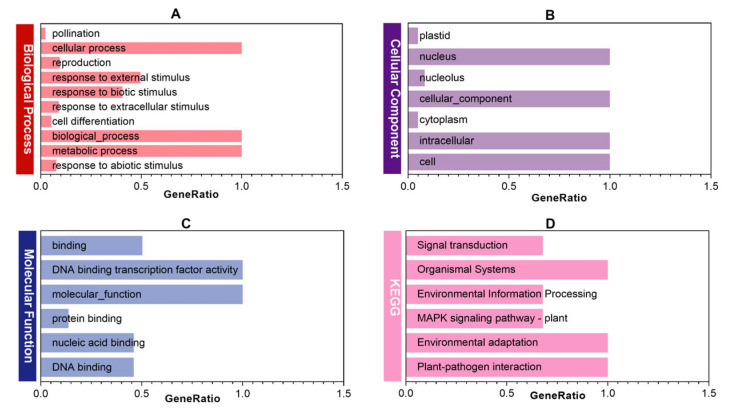
Functional annotation and enrichment analysis of *AaWRKY* genes. The figure illustrates the functional landscape of the WRKY transcription factor family in mugwort through Gene Ontology (GO) and Kyoto Encyclopedia of Genes and Genomes (KEGG) enrichment analyses. (**A**–**C**) represent Biological Process, Cellular Component, and Molecular Function in GO enrichment, respectively; (**D**) represents the KEGG enrichment results.

GO and KEGG enrichment analyses were performed on the WRKY gene of mugwort, and the findings are illustrated in Figure 8. The GO enrichment analysis revealed significant enrichment in various biological processes (Figure 8A), including responses to salicylic acid, bacteria, and chitin. Molecular functions (Figure 8C) that were prominently enriched encompassed DNA sequence binding, transcription factor binding, and cis-acting element binding. Regarding cellular components (Figure 8B), the enriched areas predominantly included the nucleus and certain cell membrane binding sites, attributed to the involvement of transcription factors. In the KEGG enrichment analysis (Figure 8D), three key pathways stood out: the MAPK signaling pathway and the plant–microbe interaction pathway.

### 2.9. RT-qPCR Validation of AaWRKY Gene Expression in Two Materials

**Figure 9:**
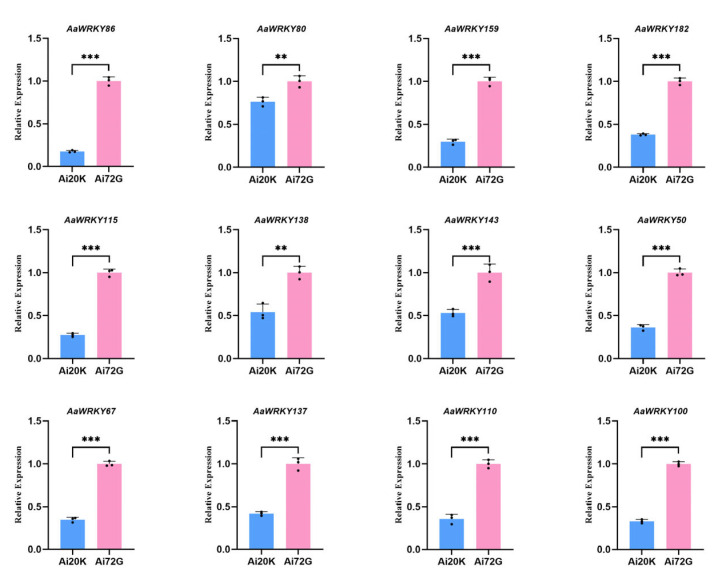
RT-qPCR validation of candidate *AaWRKY* genes in two *Artemisia argyi* materials. The blue bars represent the Ai20K group, and the pink bars represent the Ai72G group. Twelve representative genes (*AaWRKY86*, *AaWRKY80*, *AaWRKY159*, *AaWRKY182*, *AaWRKY115*, *AaWRKY138*, *AaWRKY143*, *AaWRKY50*, *AaWRKY67*, *AaWRKY137*, *AaWRKY110*, and *AaWRKY100*) were selected based on their potential roles in isoflavone biosynthesis or significant differential expression in RNA-seq. The relative expression levels were calculated using the 2^−ΔΔCt^ method, with an endogenous reference gene used for normalization. Data are shown as the mean ± standard deviation (SD) of three biological replicates. Asterisks indicate significant differences between Ai20K and Ai72G determined by Student’s *t*-test (** *p* < 0.01; *** *p* < 0.001). The expression trends of these 12 genes are highly congruent with the FPKM values obtained from RNA-seq analysis, confirming the robustness of the transcriptomic datasets. This result is the verification of the up-regulation of the WRKY gene in *Artemisia argyi* in the disease-susceptible material Ai72G.

**Figure 10:**
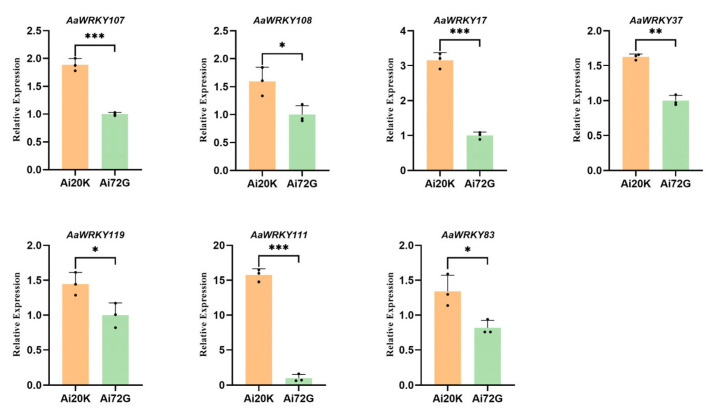
RT-qPCR validation of candidate *AaWRKY* genes in two *Artemisia argyi* materials. This figure displays the relative expression levels of seven candidate *AaWRKY* genes to validate the differential expression patterns observed in the transcriptomic data. The orange bars represent the Ai20K group, and the green bars represent the Ai72G group. The validated genes include *AaWRKY107*, *AaWRKY108*, *AaWRKY17*, *AaWRKY37*, *AaWRKY119*, *AaWRKY111*, and *AaWRKY83*. Data are shown as the mean ± standard deviation (SD) of three biological replicates. Asterisks indicate significant differences between Ai20K and Ai72G determined by Student’s t-test (* *p* < 0.05; ** *p* < 0.01; *** *p* < 0.001). The expression trends of these 7 genes are highly congruent with the FPKM values obtained from RNA-seq analysis, confirming the robustness of the transcriptomic datasets. This result is the verification of the down-regulation of the WRKY gene in *Artemisia argyi* in the disease-susceptible material Ai72G.

Based on the expression levels of the WRKY gene in two distinct mugwort materials derived from previous transcriptome data, 19 genes were selected for fluorescence quantitative PCR to validate their expression status in this study (Figure 9 and Figure 10). The results indicate that the expression levels of *AaWRKY86*, *AaWRKY80*, *AaWRKY159*, *AaWRKY182*, *AaWRKY115*, *AaWRKY138*, *AaWRKY143*, *AaWRKY50*, *AaWRKY67*, *AaWRKY137*, *AaWRKY110*, and *AaWRKY100* in disease-resistant materials were significantly lower than those in susceptible materials. Previous protein–protein interaction analyses revealed that AaWRKY110 interacts with proteins associated with the synthesis of mugwort isoflavones, suggesting that it may be a key member of the mugwort WRKY family involved in isoflavone synthesis and insect resistance. In contrast, the expression levels of *AaWRKY107*, *AaWRKY108*, AaWRKY17, *AaWRKY37*, *AaWRKY119*, *AaWRKY111*, and *AaWRKY83* in disease-resistant materials were significantly upregulated compared to those in disease-susceptible materials, indicating their potential role as positive regulatory factors in the disease resistance process of mugwort. Furthermore, there exists an interaction among WRKY family proteins, which may suggest a synergistic regulatory relationship among them. The verification of expression levels in selected representative members from a total of 182 members facilitates further experimental validation in future studies.

## 3. Discussion

In this study, we identified 182 *AaWRKY* genes across the entire genome of *Artemisia argyi*. This figure is significantly greater than that found in *Arabidopsis thaliana* (72), *Oryza sativa* (100) [32], and *Artemisia annua* (122), all of which belong to the same genus. This suggests that the WRKY family has undergone considerable expansion during the evolution of *Artemisia argyi*. The chromosomal distribution map (Figure 1) indicates a significant enrichment of *AaWRKY* genes on chromosome 4. Such a non-random distribution typically suggests the presence of “hotspots” for gene duplication in this region. Collinearity analysis further demonstrates that segmental duplication serves as the primary mechanism driving the expansion of the WRKY family in *Artemisia argyi*, as opposed to tandem duplication [33]. This finding carries important evolutionary implications: segmental duplication often entails large-scale genomic recombination and can maintain a more complete gene regulatory structure, thereby mitigating potential functional redundancy or interference associated with tandem duplication [34]. Furthermore, the majority of *AaWRKY* gene pairs display extremely low Ka/Ks ratios (<1.0), indicating that this gene family has experienced strong purifying selection pressure throughout the genome doubling and subsequent evolution of *Artemisia argyi* [35]. This evolutionary strategy ensures the precise preservation of core transcriptional regulatory functions, even as the genome expands significantly, reflecting the evolutionary resilience of *Artemisia argyi* in complex ecological niches [36].

Analysis of the physicochemical properties of 182 AaWRKY proteins revealed significant diversity and functional flexibility. Notably, up to 92.9% of these proteins were predicted to be unstable, as indicated by an instability index greater than 40, a characteristic that is not coincidental [37]. In plant signal transduction, transcription factors function as “molecular switches,” and the precise regulation of their half-lives is essential to prevent the overaccumulation of defense signals [27]. The rapid turnover of AaWRKY proteins may be mediated by the 26S proteasome pathway, which facilitates their swift degradation following responses to aphid feeding or abiotic stresses, thereby ensuring signal transience and specificity [38]. Furthermore, the isoelectric points (pI) of these proteins range from 4.78 to 10.11, indicating variability in their charge states under different cellular physiological conditions. This broad pI distribution may enable them to interact with cofactors or chromatin remodeling complexes of varying charges, allowing for precise targeting of promoter regions within the dynamic nuclear microenvironment. All members of this protein family are hydrophilic, aligning well with their functional role in transcriptional activation or repression within the aqueous environment of the nucleus.

Expression profile analysis revealed that over half of the *AaWRKY* genes exhibited high expression levels in roots (Figure 6), a characteristic closely associated with the perennial herb *Artemisia argyi*. Roots serve not only as nutrient-absorbing organs but also as sensors for underground allelochemicals and soil-borne pathogens. The significant enrichment of Group IIb members in roots indicates their potential involvement in regulating the synthesis of secondary metabolites unique to *Artemisia argyi* roots, such as specific polyacetylene compounds [39]. In the above-ground tissues, we noted the significant and specific expression of *AaWRKY11* in senescent leaves. Drawing parallels with the regulatory network of *AtWRKY53* in Arabidopsis, *AaWRKY11* may function as a trigger for leaf senescence, facilitating the transfer of nutrients from senescent tissues to new tissues by inhibiting genes associated with chloroplast development and activating genes related to protein degradation [40]. This tissue-specific expression pattern underscores the precise regulatory role of the AaWRKY family in coordinating vegetative growth and defense responses, exemplifying a typical “growth-defense trade-off” strategy [41].

WRKY transcription factors can function as both positive and negative regulators of plant responses. In rice, *OsWRKY45* is a central positive regulator of salicylic acid signaling and markedly enhances broad-spectrum resistance to *Magnaporthe oryzae* [42]. In Arabidopsis, *AtWRKY33* directly activates defensin biosynthesis, increasing resistance to *Botrytis cinerea* [23]. In several crops, including cotton (*GhWRKY34*) and wheat (*TaWRKY2*), WRKYs improve water retention and membrane stability by upregulating ABI5 or DREB genes [43,44]. WRKY proteins also participate in temperature-compensation networks; for example, the *WRKY25/26/33* co-regulatory module in Arabidopsis preserves protein homeostasis during heat stress and protects chloroplasts from thermal damage [45]. Conversely, some WRKYs act as negative regulators, for instance, *AtWRKY7*, preventing excessive defense activation that would otherwise impede normal growth [46]. This bidirectional regulation enables plants to allocate limited resources precisely when coping with sudden external stresses.

The biosynthesis of isoflavones in *Artemisia argyi* is governed by a complex transcriptional network. The protein–protein interaction (PPI) network constructed in this study (Figure 5) identified AaWRKY110 as a central hub, capable of interacting with several rate-limiting enzymes, including AaPAL, Aa4CL, and AaCHS8. This finding not only supports the established notion that WRKY proteins directly bind to the W-box *cis*-element but also suggests that AaWRKY110 may function as a molecular scaffold, facilitating the formation of metabolons and thereby enhancing metabolic flux efficiency [47]. KEGG enrichment analysis prominently highlighted the MAPK signaling pathway (Figure 8), offering a novel perspective on the metabolic regulation of *Artemisia argyi*. In typical stress responses, the upstream MAPK cascade activates WRKY transcription factors via phosphorylation, which subsequently triggers the production of secondary metabolites [48]. As a member of Group I, AaWRKY110 possesses multiple potential phosphorylation sites and is likely to serve as a downstream effector of the MAPK cascade, translating environmental stress signals into chemical defense instructions for isoflavone synthesis [49].

Through the transcriptome comparison between resistant and susceptible materials (Ai20K vs. Ai72G), we deeply revealed the differences in the insect-resistance mechanisms mediated by AaWRKY. In the resistant variety Ai20K, members of Subgroup IIa and III exhibited relatively high basal expression levels. This priming state enables plants to rapidly form a chemical defense barrier at the early stage of aphid invasion, similar to the salicylic acid (SA)-mediated systemic acquired resistance (SAR) in model organisms [50]. In contrast, in the susceptible variety Ai72G, although genes such as *AaWRKY110* were significantly up-regulated after infection, it was more like a delayed burst after damage. At this time, the pests had already completed colonization, and the plant’s defense response became ineffective due to the lack of timeliness [51]. In particular, the specific high expression of *AaWRKY82* and *AaWRKY71* in the resistant variety makes them preferred candidate genes for improving the resistance of *Artemisia argyi*. These genes may finely regulate the specific immunity of *Artemisia argyi* against piercing sucking pests by mediating the crosstalk between SA and jasmonic acid (JA) signaling pathways.

## 4. Materials and Methods

### 4.1. Identification and Analysis of Physicochemical Properties of WRKY Genes in Artemisia argyi

All protein sequences and the corresponding GFF3 annotation files were obtained from the whole-genome database of Artemisia argyi. The hidden Markov model of the WRKY domain (Pfam ID: PF03106) was utilized as a search seed in HMMER v3.0 [52] to conduct a preliminary screening of the Artemisia argyi whole-genome protein database, with an E-value threshold typically set at 1 × 10^−5^. To ensure the accuracy of the identification results, all candidate protein sequences were submitted to the NCBI-CDD and SMART [53] databases for verification of domain integrity. The physicochemical properties of 182 proteins were analyzed using TBtools (v2.420) [54]. Based on the GFF3 annotation information of the Artemisia argyi genome, the start and end position information of 182 AaWRKY genes were extracted. Subsequently, these physical positions were annotated on the 17 main chromosomes and 7 unanchored contigs of Artemisia argyi using TBtools (v2.420) to analyze their distribution characteristics within the genome.

### 4.2. Systematic and Phylogenetic Analysis of WRKY Proteins in Artemisia argyi

To elucidate the evolutionary relationships of WRKY transcription factors in *Artemisia argyi*, this study performed a phylogenetic analysis of 182 identified AaWRKY protein sequences alongside the WRKY members of the model plant *Arabidopsis thaliana*. Multiple sequence alignments of full-length protein sequences were conducted using Mafft version 6 [55] software, emphasizing the highly conserved WRKY domain and its adjacent sequences. The MEGA (v11.0) [56] software facilitated the construction of the molecular evolutionary tree, employing the Maximum Likelihood (ML) method based on the optimal amino acid substitution model. To assess the stability of evolutionary branches, 2000 bootstrap replicates were executed. Based on the established classification criteria for the Arabidopsis WRKY family and the characteristics of the zinc-finger motif in AaWRKY proteins, the 182 members were categorized into three major groups: Group I, Group II (which includes subgroups a, b, c, d, and e), and Group III. The tvBOT [57] tool was utilized to enhance the phylogenetic tree, incorporating background color, species identifiers, and subfamily labels.

### 4.3. Collinearity Analysis of WRKY Genes in Artemisia argyi

To elucidate the expansion mechanism of the WRKY gene family in Artemisia argyi during evolution, this study conducted an intra-genomic collinearity analysis. First, the Blastp v2.13.0 software was used to perform an all-against-all alignment of protein sequences across the entire Artemisia argyi genome with an E-value threshold of 1 × 10^−5^ to obtain information on potential homologous gene pairs. The alignment results and the GFF3 annotation file of the Artemisia argyi genome were used as inputs, and the MCScanX v1.0.0 [58] software was employed to search for collinear regions within the genome. The criterion for determining syntenic blocks was generally set to contain at least 5 collinear gene pairs. The TBtools software v2.423 was used to extract collinear pairs involving 182 AaWRKY members from the whole-genome collinearity results and distinguish their duplication patterns. Mafft was used to perform protein sequence alignment of the identified collinear gene pairs, which were then converted into corresponding codon-aligned sequences. The Ka/Ks calculation module in KaKs_Calculator 2.0 [59] was used with the YN00 method to calculate the non-synonymous substitution rate (Ka), synonymous substitution rate (Ks), and their ratio (Ka/Ks). The selection pressure on genes was judged based on the Ka/Ks ratio. The TBtools (Advanced Circos) was used to draw an intra-genomic collinearity map of Artemisia argyi, where 182 AaWRKY genes were anchored on 17 chromosomes, and the collinear relationships between genes were shown by connecting lines.

### 4.4. Collinearity Analysis of WRKY Genes in Artemisia argyi and Other Plants

To examine evolutionary conservation and phylogenetic relationships of the *AaWRKY* gene family across species, we performed a cross-species collinearity analysis involving *Artemisia argyi*, the model plant *Arabidopsis thaliana*, and the closely related *Chrysanthemum morifolium*. Whole-genome protein sequences and GFF3 annotation files for A. thaliana and C. morifolium were retrieved from public databases. One-to-one protein alignments for Aa versus At and Aa versus Cm were generated with Diamond, using an E-value cutoff of 1 × 10^−5^. We then used the JCVI (Python) toolkit v3.12 to identify collinear regions by combining alignment outputs with each species’ GFF3 annotations. Homologous pairs that included WRKY family members were extracted from the genome-wide collinearity results using TBtools’ file-screening utility. Finally, a cross-species collinearity map was produced with TBtools (Multiple Synteny Plotter).

### 4.5. Interaction Analysis of WRKY Proteins in Artemisia argyi

To predict potential biological functions of WRKY proteins in Artemisia argyi and their roles in regulating secondary metabolism, we constructed a protein–protein interaction network using the STRING database. Full-length sequences of the 182 AaWRKY proteins identified in this study served as the primary input. Guided by the correlation analysis between the Artemisia argyi transcriptome and metabolome, we selected 10 key enzymes involved in isoflavone biosynthesis: Aa4CL, AaANS2, AaC4H, AaCHI2A, AaCHS8, AaDFR1, AaFLS1, AaIFS2, AaPAL, and AaI2H. From the initial network, we identified 54 AaWRKY proteins that participate in interactions; these 54 members span all seven subgroups. We visualized and refined the STRING output in Cytoscape (v3.9.1) and used the cytoHubba plugin to compute node degree and closeness centrality to pinpoint core hubs. We then examined WRKY members with high connectivity to the isoflavone-biosynthetic enzymes, with particular attention to the core hub AaWRKY110.

### 4.6. Expression Analysis of WRKY Genes in Different Tissue Parts of Artemisia argyi

This study aimed to analyze the gene expression patterns of Artemisia argyi WRKY genes across various tissue types and time intervals. The Artemisia argyi WRKY gene sequences were extracted from the gene expression dataset available on the Integrated Medicinal Plantomics (IMP) website. Subsequently, the expression levels of these genes were utilized to generate a visual representation in the form of a heat map using the heat map plotting feature of the TBtools software v2.423.

### 4.7. Transcriptome Analysis and WRKY Gene Expression Analysis of Ai20K and Ai72G

Two Artemisia argyi materials, Ai20K and Ai72G, were selected, with three biological replicates per material, yielding six samples for transcriptome sequencing. Raw reads from the Illumina platform were filtered with fastp to remove adapter-containing and low-quality reads, producing high-quality clean data. Clean reads were aligned to the Artemisia argyi reference genome with HISAT2 to determine their chromosomal positions. Read counts per gene were obtained using featureCounts, and expression levels were normalized by FPKM (Fragments Per Kilobase of transcript per Million mapped reads). Differentially expressed genes (DEGs) were identified using thresholds of |log_2_(Fold Change)| ≥ 1 and *P*-adj < 0.05. Using the sequence IDs of 182 previously identified AaWRKY genes, we extracted their FPKM values from the genome-wide expression matrix with a script to create an AaWRKY family-specific expression matrix, which was visualized as a heatmap in TBtools.

### 4.8. GO and KEGG Enrichment Analysis of WRKY Genes in Artemisia argyi

To characterize the functions and pathway involvement of the 182 AaWRKY genes, we performed comprehensive functional annotation and enrichment analyses. We annotated the 182 AaWRKY protein sequences against the Gene Ontology (GO) database using EggNOG-mapper v2.1 and categorized the results into Biological Process (BP), Molecular Function (MF), and Cellular Component (CC). We mapped the sequences to the Kyoto Encyclopedia of Genes and Genomes (KEGG) using KOBAS 3.0 to identify associated metabolic and signaling pathways. The full set of protein-coding genes from the Artemisia argyi genome served as the background reference. We assessed the significance of each GO term and KEGG pathway using the hypergeometric test and corrected original *p*-values for multiple testing with the Benjamini–Hochberg method to obtain false discovery rate (FDR) values. Enrichment was considered significant at FDR < 0.05. Finally, we visualized the enrichment results using TBtools.

### 4.9. Acquisition of Plant Materials and Fluorescence Quantitative PCR Detection of WRKY Genes

Two varieties of mugwort were planted in the experimental field of Henan Academy of Agricultural Sciences. Among them, Ai72G is a material without resistance to aphids, while Ai20K is a material with resistance to aphids. After natural pest occurrence, the leaves were quickly frozen and stored in a −80 °C refrigerator. Subsequently, transcriptome sequencing was conducted. Real-time fluorescent quantitative PCR was performed using a Step One Plus PCR system (Thermo Fisher Scientific, Waltham, MA, USA). The EasyScript^®^ First-Strand cDNA Synthesis Super Mix reagent kit (TransGen Biotech, Beijing, China) was used to synthesize first-strand cDNA, and RT-qPCR was performed using a TransStart^®^ Top Green qPCR Super Mix kit (TransGen Biotech, China) with 18S rRNA as a reference gene. The total PCR volume was 20 μL. The experimental procedure consisted of pre-denaturation at 95 °C for 30 s, followed by 40 cycles at 95 °C for 5 s, 58 °C for 15 s, and 72 °C for 10 s. The experiment was repeated three times, and 2^−ΔΔCt^ was used to calculate relative expression [60]. Gene primer sequences are shown in Table S3.

## 5. Conclusions

In this study, a comprehensive genome-wide identification of the 182 *AaWRKY* gene members in *Artemisia argyi* was performed. Evolutionary analysis revealed that segmental duplication was the primary driver of family expansion, while the family remained under strong purifying selection, ensuring high functional conservation. Phylogenetic and physicochemical analyses confirmed that AaWRKY proteins possess typical transcription factor characteristics and coordinate plant growth through subgroup-specific functional differentiation. The research further highlighted the pivotal role of *AaWRKYs* in tissue development and secondary metabolism. More than half of the *AaWRKY* genes exhibited significant root enrichment, while the protein–protein interaction network centered on AaWRKY110 was found to mediate the transcriptional regulation of the isoflavonoid biosynthetic pathway, suggesting a decisive influence on the accumulation of medicinal components. Regarding biotic stress, comparative transcriptomic analysis revealed a distinct “priming” mechanism in the resistant cultivar Ai20K, where Subgroup IIa and III members (*AaWRKY82*, *108*, and *128*) maintained high basal expression levels. Conversely, the susceptible cultivar showed a delayed and ineffective stress response. In summary, this study clarifies the evolutionary and regulatory framework of the *AaWRKY* family and identifies key candidates for the synergistic regulation of isoflavonoid accumulation and aphid resistance, providing theoretical foundations and genetic resources for breeding high-yield and resilient *Artemisia argyi* cultivars.

## Data Availability

The original contributions presented in the study are included in the article/Supplementary Material; further inquiries can be directed to the corresponding authors.

## Supplementary Materials

The following supporting information can be downloaded at https://www.mdpi.com/article/10.3390/ijms27072981/s1.
