# Supplementary material for: Genome-Wide Identification of WRKY Gene Family in Artemisia and Its Expression Analysis of Aphid Resistance
Source: Int J Mol Sci. 2026 Mar 25;27(7):2981. doi: 10.3390/ijms27072981 (PMC13073117; doi:10.3390/ijms27072981)
Supplement: Supplementary file 1 [file ijms-27-02981-s001.zip › FigureS1_Physical and chemical properties.pdf]

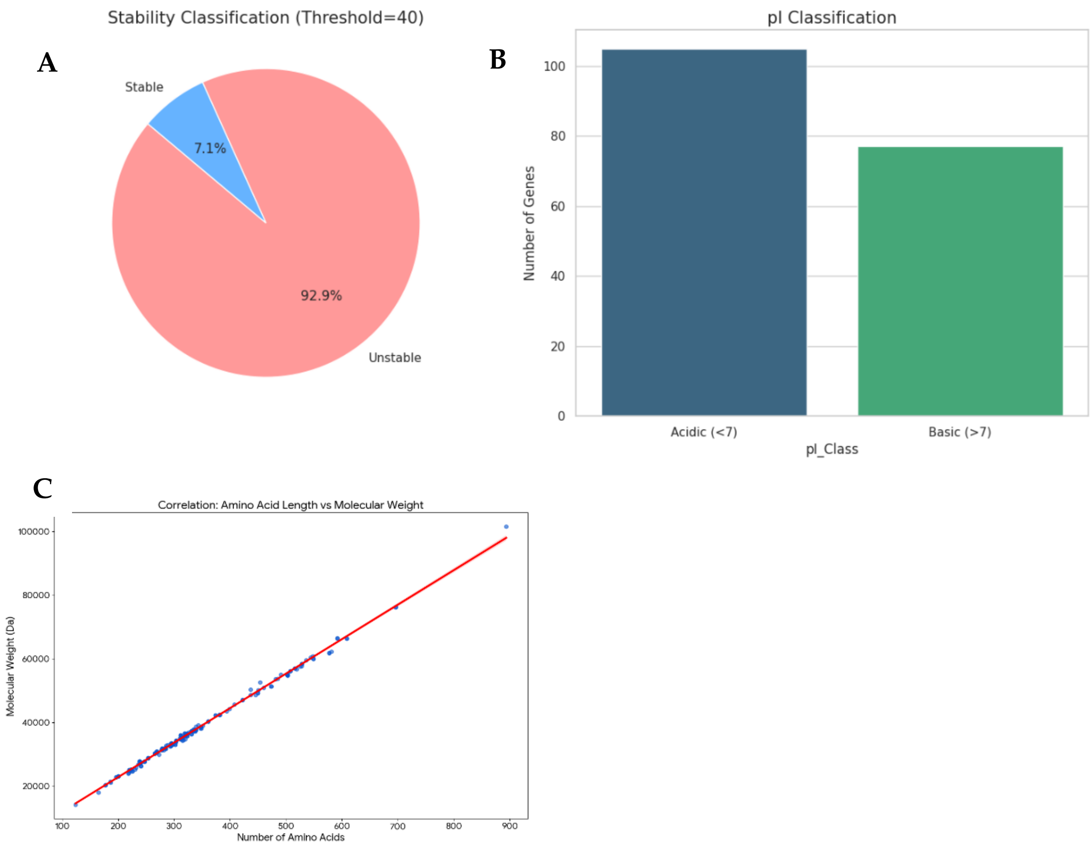

FigureS1. Physical and chemical properties of AaWRKYs. A.Pie chart illustrating the proportion of stable (instability index < 40) and unstable (instability index > 40) AaWRKY proteins; B.Bar chart showing the number of acidic (pI < 7.0) and basic (pI > 7.0) members within the AaWRKY family. C. Scatter plot with a linear regression line (red) showing the relationship between the number of amino acids and the molecular weight (Da) for all 182 AaWRKY proteins. The shaded area represents the 95% confidence interval.
