# Supplementary material for: Genome-Wide Identification of WRKY Gene Family in Artemisia and Its Expression Analysis of Aphid Resistance
Source: Int J Mol Sci. 2026 Mar 25;27(7):2981. doi: 10.3390/ijms27072981 (PMC13073117; doi:10.3390/ijms27072981)
Supplement: Supplementary file 1 [file ijms-27-02981-s001.zip › FigureS3_RNAseq.pdf]

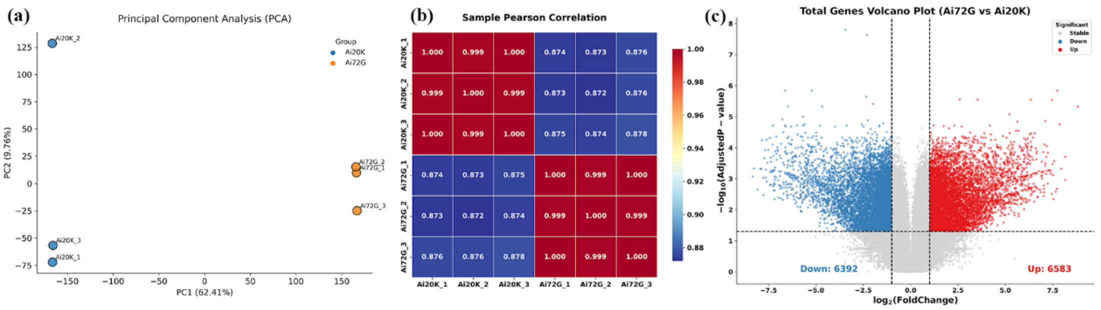

FigureS3. Sample evaluation and differential expression analysis of transcriptome data. (a) Principal Component Analysis (PCA); (b) Sample Pearson Correlation Heatmap; (c) Volcano Plot of Differentially Expressed Genes (DEGs)
