# Supplementary figures and images for: Genome-Wide Identification of WRKY Gene Family in Artemisia and Its Expression Analysis of Aphid Resistance
Source: Int J Mol Sci. 2026 Mar 25;27(7):2981. doi: 10.3390/ijms27072981 (PMC13073117; doi:10.3390/ijms27072981)

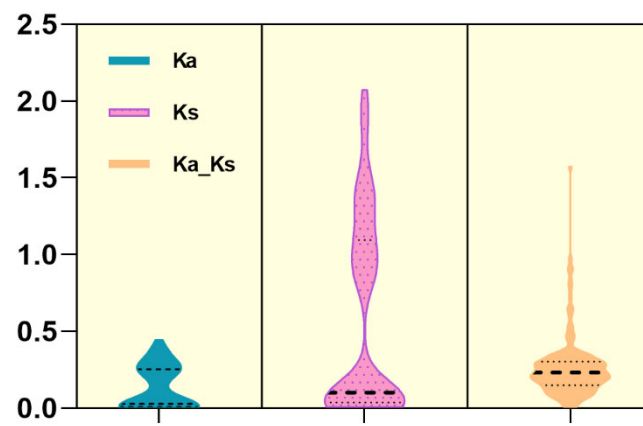

Figure S2. Distribution of Ka, Ks, and Ka/Ks ratios for *Artemisia argyi* gene pairs.

Supplement: Supplementary file 1 [file ijms-27-02981-s001.zip › FigureS2.pdf]
